# Down-regulation of microRNA-34a-5p promotes trophoblast cell migration and invasion via targetting Smad4

**DOI:** 10.1042/BSR20181631

**Published:** 2019-02-12

**Authors:** Fang Xue, Jing Yang, Qirong Li, Haibin Zhou

**Affiliations:** 1 Department of Gynaecology and Obstetrics, Jinan Maternal and Child Health Hospital, Jinan, Shandong 250012, P.R. China; 2 Department of Gynaecology and Obstetrics, Qilu Hospital of Shandong University, Jinan, Shandong 250012, P.R. China

**Keywords:** HTR-8/SVneo cells, MicroRNA-34a-5p, migration and invasion, Smad4 signaling pathway

## Abstract

Trophoblastic dysfunction, such as insufficient migration and invasion, is well-known to be correlated with preeclampsia (PE). Recently, microRNAs (miRNAs) have been implicated in diverse biological processes and human diseases, including PE. However, the expression and functions of miRNAs in the progression of PE, especially in the regulation of trophoblast cell migration and invasion remain largely unclear. Here, we compared the miRNAs expression profiles of PE patients with healthy controls using microarray assay and chose a significant increased miRNA-*miR-34a-5p* for further investigation. Overexpression of *miR-34a-5p* dramatically reduced migration and invasion in trophoblast HTR-8/SVneo cells, whereas enhanced by its inhibitor. Luciferase activity assay showed that *miR-34a-5p* directly target Smad family member 4 (Smad4), which is associated with cancer cell invasiveness and metastasis. We also found that Smad4 was down-regulated in PE patients, and an inverse relationship between Smad4 and *miR-34a-5p* expression levels was observed in placental tissues from PE patients. Further study showed that knockdown of Smad4 effectively attenuated the promoting effects of *miR-34a-5p* inhibition on the migration and invasion of HTR-8/SVneo cells. Taken together, these findings suggest that inhibition of *miR-34a-5p* improves invasion and migration of trophoblast cells by directly targetting Smad4, which indicated the potential of *miR-34a-5p* as a therapeutic target against PE.

## Introduction

Preeclampsia (PE) is a significant pregnancy complication that occurs only after 20 weeks of gestation and characterized by proteinuria and hypertension [1]. It has been estimated that PE affected about 3–5% pregnancies worldwide and is a major cause of the neonatal and maternal morbidity and mortality [2–4]. Although the exact mechanism underlying PE remains largely elusive, it is believed that trophoblastic dysfunction, such as inadequate migration and invasion is central to the pathogenesis of PE [5,6]. Therefore, a better understanding of the regulation of human trophoblast invasion, as well as the underlying molecular mechanisms, will improve the diagnosis and treatment of PE.

MicroRNAs (miRNAs) are small non-coding RNAs of approximately 22 nucleotides that can regulate the expression of their target genes through binding to the 3′ untranslated regions (UTRs) of the mRNAs [7]. Increasing evidence has indicated the involvement of miRNAs in the regulation of trophoblast motility and invasiveness [8,9]. For example, Jiang et al. found that *miR-520g* was up-regulated in patients with severe PE, and overexpression of *miR-520g* significantly suppressed the invasion of HTR-8/SVneo cells via at least partial inhibition of matrix metalloproteinase 2 (MMP2) [10]. Xiao et al. showed that miR-144 was down-regulated in placentas of the patients with PE and associated with increased invasive ability of trophoblastic cells [11]. Kim et al. demonstrated that miR-31-5p elicited endothelial dysfunction associated with PE via post-transcriptional down-regulation of eNOS [12]. In addition, several miRNAs have been considered as promising circulating biomarkers in early detection of PE [13]. Therefore, further understanding of the miRNA aberrantly expressed in PE patients is beneficial for understanding the pathogenesis and progression of PE, thereby proposing more therapeutic interventions.

In the present study, we quantified the expression profiles of miRNAs in placentas from PE and normal pregnancies, and further investigated the effect and regulatory mechanisms of *miR-34a-5p* on cell invasion and migration of HTR-8/SVneo cells. Our findings may provide new insights into the mechanisms underlying the regulation of trophoblast function and the pathogenesis of PE.

## Materials and methods

### Patients and samples collection

Placental tissues from 20 women with severe PE were collected after cesarean section at the Department of Gynaecology and Obstetrics, Qilu Hospital of Shandong University between March 2015 and May 2016. Severe PE was strictly defined according to the definition in Williams Obstetrics (23rd edition). Briefly, patients had new-onset systolic blood pressure (SBP) ≥160 mmHg or diastolic blood pressure (DBP) ≥110 mmHg on two or more occasions, accompanying severe proteinuria (2.0 g per 24 h or greater than 2+ by dipstick) during pregnancy. For the control group, women with renal disease, cardiovascular disease, transient hypertension in pregnancy, gestational diabetes mellitus, hepatitis, any evidence of spontaneous abortion, intrauterine fetal death, fetal chromosomal or other pregnancy complications were excluded from the present study. The present study was approved by the Research Ethics Committee of Qilu Hospital of Shandong University. Informed consents were obtained from all patients. A total of 20 women who were healthy and pregnant were recruited as the control group. Placental tissues and serum samples were stored frozen at 20°C until analyzed. Venous blood samples of study subjects were collected in EDTA tubes (5 ml) and immediately centrifuged at 1000 ***g*** for 10 min to collect plasma, which was stored at −40°C until use. Three placental tissues samples of each group were selected for the miRNA microarray analysis. All of the patients in our study were primiparas and the general clinical data, such as age, gestational week, infant birth weight, etc., were matched between groups (Table 1).

**Table 1:** Clinical parameters of patients enrolled in our study

	Control (*n*=20)	PE (*n*=20)	*P*-value
Age (years)	27.00 ± 0.68	28.55 ± 0.83	0.1547
SBP (mmHg)	115.03 ± 2.31	166.72 ± 1.42	<0.01
DBP (mmHg)	71.72 ± 1.31	105.31 ± 1.67	<0.01
Proteinuria (g/24 h)	NA	3.46 ± 0.31	NA
Nulliparous (%)	90	95	NA
Gestational day at delivery (days)	273.1 ± 2.68	253.4 ± 2.31	<0.01
Infant birth weight (g)	3213 ± 112.37	2351 ± 80.22	<0.01

Data are shown as mean ± S.D., and significant difference between Control and PE patients are analyzed with Student’s *t*-test. Abbreviations: SBP, systolic blood pressure; DBP, diastolic blood pressure; NA, not available.

### Microarray assay

Total RNA in the placenta was isolated using mirVana miRNA Isolation Kit (Life Technologies) according to the manufacturer’s instructions. Quantity and quality were assessed with NanoDrop 2000 Spectrophotometer and Agilent 2100 Bioanalyze. One microgram of total RNA was used as the input for the labeling reaction and hybridized to analyze the Affymetrix GeneChip^®^ miRNA 3.0 Array containing 5639 human targets. Arrays were scanned using a Genechip Array scanner 3000 7G (Affymetrix). The image analysis was carried out using the ImaGene^®^ 9 (miRCURY LNA™ microRNA Array Analysis Software, Exiqon). The raw intensity data were further analyzed using R software.

### Quantitative real-time PCR

The gene expression levels of *miR-34a-5p* and Smad4 in HTR-8/SVneo cells, as well as clinical PE tissues, were measured by quantitative real-time PCR (qRT-PCR) on an ABI PRISM 7500 Fluorescent Quantitative PCR System (Applied Biosystems, Foster City, CA, U.S.A.). RNA extraction was performed using TRIzol reagent (Takara, Qingdao, China). For miRNA reverse transcription, cDNA was synthesized using the SuperScript II kit (Invitrogen) according to the manufacturer’s instructions. For mRNA reverse transcription, cDNA was synthesized using PrimeScript RT Reagent Kit with gDNA Eraser (TaKaRa Bio Inc., Shiga, JP). Relative quantification was determined by normalization to U6 or GAPDH. Real-time PCR primers used for *miR-34a-5p* forward: 5′-CCCACATTTCCTTCTTATCAACAG-3′; reverse: 5′-GGCATCTCTCGCTTCATCTT-3′. U6 forward: 5′-TGCGGGTGCTCGCTTCGCAGC-3′; reverse: 5′-CCAGTGCAGGGTCCGAGGT-3′. Smad4 forward: 5′-CGGGATCCCGATGGACAATATGTCTATTACG-3′; reverse: 5′-GGATCCTCAGTCTAAAGGTTGTGGG-3′. GAPDH forward: 5′-AGGTCGGTGTGAACGGATTTG-3′, reverse: 5′-TGTAGACCATGTAGTTGAGGTCA-3′. The qRT-PCR assays were performed in triplicate and the change in expression level was calculated using the 2^−ΔΔ*C*^_t_ method.

### Cell culture

HTR-8/SVneo cells (human placenta tropholast cell line; American Type Culture Collection (ATCC), Manassas, VA) were cultured in Dulbecco’s modified Eagle’s medium/F12 (DMEM/F12, Sigma, St. Louis, MO) containing 100 U/ml penicillin and streptomycin antibiotics, 10% fetal bovine serum (FBS). The cells were cultured at 37°C in an incubator buffered with 5% carbon dioxide and 95% air. The cells were passaged when they reached 90% confluence.

### Transfection

*miR-34a-5p* mimics, mimics negative control (NC), *miR-34a-5p* inhibitor and inhibitor NC were purchased from GenePharma (Shanghai, China). The sequences are as follows: *miR-34a-5p* mimics, sense 5′-UGGCAGUGUCUUAGCUGGUUGU-3′ antisense 5′-ACAACCAGCUAAGACACUGCCA-3′; mimics NC, sense 5′-UUCUCCGAACGUGUCACGUTT-3′ antisense 5′-ACGUGACACGUUCGGAGAATT-3′; *miR-34a-5p* inhibitor, 5′-ACAACCAGCUAAGACACUGCCA-3′; inhibitor NC 5′‐CAGUACUUUGUGUAGUACAA-3′. si-Smad4-1, si-Smad4-2, si-Smad4-3, and scramble siRNA were also synthesized and provided by GenePharma (Shanghai, China). The sequences are as follows: si-Smad4-1, 5′-AGGACAGCAGCAGAATGGATTTACT-3′; si-Smad4-2, 5′-GGTCAGCCAGCTACTTACCATCATA-3′; si-Smad4-3, 5′-CATACACACCTAATTTGCCTCACCA-3′; si-Scramble, 5′-AGGCGACGAAGAGTATAGTTACACT-3′. Transfection of cells with miRNAs and si-Smad4 was performed using Lipofectamine 2000 Reagent (Invitrogen). Cells were harvested 48 h after transfection for further studies.

### Invasion and migration assays

Transwell invasion assays were measured using 8 mm membrane pores transwell chambers (Corning, New York, U.S.A.). 2 × 10^4^ cells in culture medium without FBS were seeded into the upper chamber pre-coated with Matrigel (BD, Bedford, MA, U.S.A.). After incubation for 24 h, cells that migrated onto the lower surface of the membrane were quickly fixed by 70% methanol and stained with 0.2% crystal violet. Then the non-invading cells on the upper membrane surface were removed with cotton swabs. Invaded cells were counted and photographed using an Eclipse TE2000-S inverted microscope (Nikon, Japan).

For migration analysis, the transfected cells were grown for 24 h, and a wound was then generated using a 100 μl pipette tip. A virtual microscope (BX51; Olympus) was used to measure the wound width at 24 h. Image analysis was performed using ImageJ software (NIH). The experiment was performed three times.

### Immunofluorescence

After 24 h the transfection, the cells were fixed in absolute ethyl alcohol for 15 min at room temperature, washed twice with PBS. Fixed cells were stained with primary antibody, anti-Smad4 (Cat no. ab40759, 1:200 dilutions, Abcam, Cambridge, MA, U.S.A.) for 1 h at room temperature, followed by incubation with secondary antibody conjugated with FITC. DAPI (0.1 μg/ml) was added to the secondary antibody mixture to visualize nuclei. Fluorescence images were collected and analyzed using an inverted fluorescence microscope.

### Luciferase reporter assay

3′-UTR of Smad4 and the mutated sequence were inserted into the pGL3 control vector (Promega Corporation, Madison, WI, U.S.A.) to construct wt Smad4-3′-UTR vector and mutant Smad4-3′-UTR vector, respectively. For luciferase reporter assay, HTR-8/SVneo cells were transfected with the corresponding vectors; 48 h after transfection, the dual-luciferase reporter assay system (Promega, Shanghai, the People’s Republic of China) were used to measure the luciferase activity. All experiments were performed in triplicate.

### Western Blot

Total protein was extracted using radio immunoprecipitation assay (RIPA) lysis buffer (Beyotime Biotechnology, Shanghai, China). Concentrations of total cellular protein were determined using a BCA assay kit (Pierce, Rockford, IL, U.S.A.). Total protein samples (30 μg) were analyzed by 12% SDS/PAGE gel and transferred to PVDF membranes (GE Healthcare, Freiburg, DE) by electroblotting. Primary antibodies against Smad4 (Cat no. ab40759, 1:2,000 dilution, Abcam, Cambridge, MA, U.S.A.) and β-actin (Cat no. sc-47778, 1:2000 dilution, Santa Cruz Biotechnology,) were probed with proteins on the membrane at 4°C overnight. After incubating with secondary antibodies (Cat no. #5946, 1:10000 dilution, Cell Signaling Technology, Danvers, MA), Bands were detected by enhanced chemiluminescence (ECL) kit (GE Healthcare, Freiburg, DE). The intensity of the bands of interest was analyzed by Image J software (Rawak Software, Inc. Munich, Germany).

### Statistical analysis

Statistical analysis was performed using the SPSS program (version 18.0; SPSS, Chicago, IL, U.S.A.). Data were presented as mean ± S.D. Student’s *t*-test or one-way ANOVA were used to analyze the difference among between sample groups. Pearson’s or Spearman’s analysis was used in correlation analysis. *P*≤0.05 was considered as statistically significant.

## Results

### *miR-34a-5p* was up-regulated in PE clinical samples

**Figure 1:**
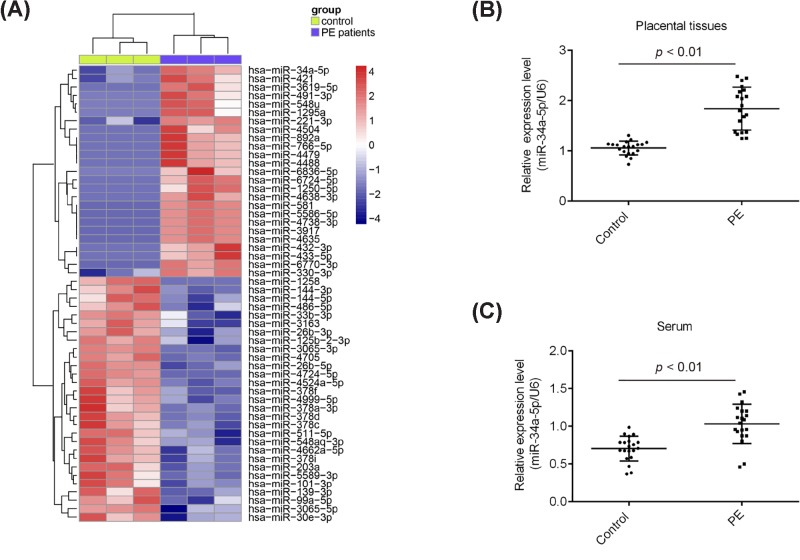
*miR-34a-5p* was up-regulated in preeclamptic placenta specimens (**A**) Heatmap of normalized expression levels of miRNAs in placentas samples from three patients with PE and three women with normal pregnancies. Blue indicates low expression levels; red indicates high expression levels. (**B**) Real-time PCR was performed to determine the expression levels of *miR-34a-5p* in preeclamptic placentas (*n*=20) and normal pregnancies (*n*=20), as well as in (**C**) serum samples from preeclamptic placentas (*n*=20) and normal pregnancies (*n*=20). *P*<0.01 vs. Control group.

To explore the potential role of miRNAs in PE, we performed miRNA microarray in three placental tissues from women with severe PE and three placental tissues from women who were healthy and pregnant. The miRNA microarray identified 25 miRNAs were up-regulated and 29 miRNAs were down-regulated in PE patients, compared with those in control group (Figure 1A). Among them, *miR-34a-5p* is one of the most significantly up-regulated miRNAs in placental tissues and many studies have been shown the involvement of *miR-34a-5p* in the regulation of cancer cell invasion and migration [14,15]. Given these, we chose *miR-34a-5p* for further study. To further confirm the microarray results, we performed qRT-PCR to detect the expression of *miR-34a-5p* between normal and severe PE placentas. In comparison with the normal placentas, *miR-34a-5p* was significantly up-regulated in severe PE placentas (Figure 1B). Moreover, the expression levels of *miR-34a-5p* were remarkably increased in serum samples from PE patients compared with healthy examined individuals (Figure 1C). Collectively, these results indicated that *miR-34a-5p* may be a novel factor associated with the development of PE.

### miR-34a-5p inhibitor enhanced the invasion of trophoblast cells

**Figure 2:**
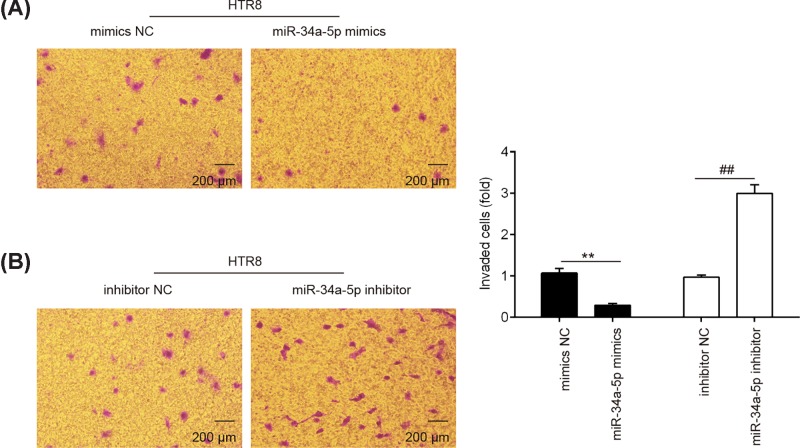
Down-regulation of *miR-34a-5p* promotes the invasion of trophoblast cells (**A** and **B**) Representives of HTR-8/SVneo cell invasion determined with transwell invasion assays (magnification 200×). Cells were transfected with *miR-34a-5p* mimics, *miR-34a-5p* inhibitor or miRNA negative controls as indicated. Data represent the mean ± S.D. of three independent experiments. ^**^P < 0.01 vs. mimics NC; ^##^*P*< 0.01 vs. inhibitor NC.

Since HTR8/SVneo is a widely-used first trimester trophoblast cell line [16], we used it as a model to study the role of *miR-34a-5p* in the invasiveness of trophoblast cells. HTR8/SVneo cells were transfected with *miR-34a-5p* mimics or its inhibitor to overexpress or knockdown the expression of *miR-34a-5p*. Next, transwell invasion assay were used to determine the invasiveness of trophoblastic cells. As shown in Figure 2A, overexpression of m*iR-34a-5p* significantly inhibited the invasion of HTR8/SVneo cells compared with the scramble controls. In contrast, the invasion of HTR8/SVneo cells that were transfected with *miR-34a-5p* inhibitor was enhanced (Figure 2B). These results clearly suggested that down-regulation of *miR-34a-5p* could enhance the invasion of trophoblast cells.

### miR-34a-5p inhibitor promoted the migration of trophoblast cells

**Figure 3:**
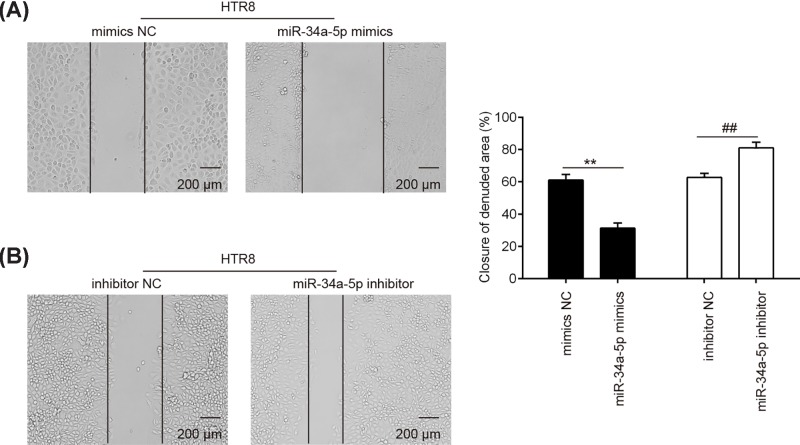
Down-regulation of *miR-34a-5p* promotes the migration of trophoblast cells HTR-8/SVneo cells were transfected with *miR-34a-5p* mimics, *miR-34a-5p* inhibitor or miRNA negative controls. (**A** and **B**) Cell migration was measured using the wound healing assay. Data represent the mean ± S.D. of three independent experiments. ^**^P < 0.01 vs. mimics NC; ^##^*P* < 0.01 vs. inhibitor NC.

Next, we assessed the effect of *miR-34a-5p* on the migration of trophoblast cells by using wound healing assay in HTR8/SVneo cells. As expected, overexpression of *miR-34a-5p* significantly inhibited the migration of HTR8/SVneo cells compared with the scramble controls (Figure 3A). In contrast, inhibition of *miR-34a-5p* by its special inhibitor obviously enhanced the migration of HTR8/SVneo cells (Figure 3B). These data indicate that down-regulation of *miR-34a-5p* could promote the migration of trophoblast cells.

### Smad4 was a direct target of *miR-34a-5p*

**Figure 4:**
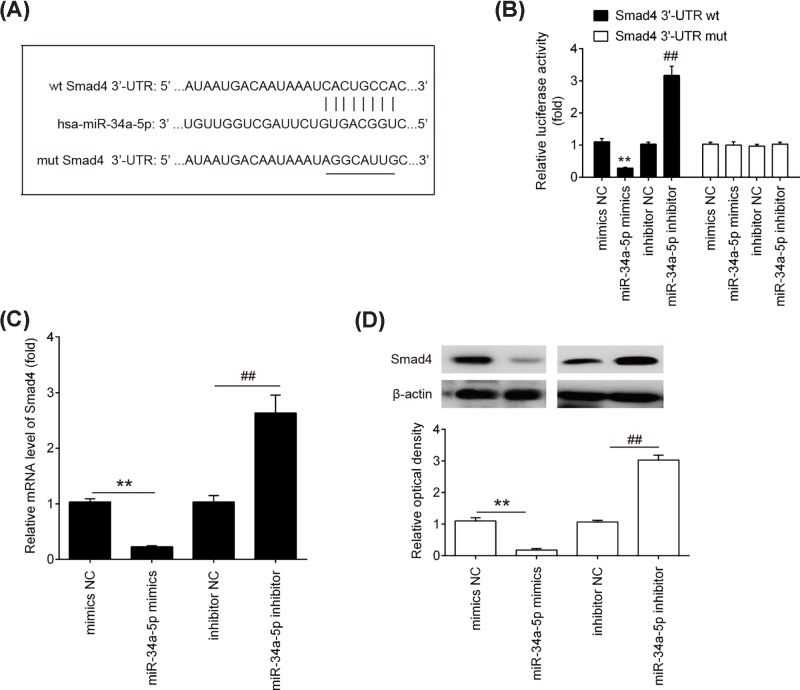
Smad4 is a direct target of *miR-34a-5p* (**A**) The putative binding site of *miR-34a-5p* and Smad4 is shown. (**B**) Luciferase assay of HTR-8/SVneo cells co-transfected with firefly luciferase constructs containing the Smad4 wild-type or mutated 3′-UTRs and miR-34a-5p mimics, mimics NC, miR-34a-5p inhibitor or inhibitor NC, as indicated (*n*=3). (**C** and **D**) The expression of Smad4 mRNA and protein after transfection with *miR-34a-5p* mimics or *miR-34a-5p* inhibitor was measured by qRT-PCR and Western Blot. Data represent the mean ± S.D. of three independent experiments. ^**^P<0.01 vs. mimics NC; ^##^*P*<0.01 vs. inhibitor NC.

To further investigate the underlying mechanism by which down-regulation of *miR-34a-5p* functions in the improvement of the invasion and migration of trophoblast cells, we first performed bioinformatics analysis using the webservers Targetscan, miRanda and PicTar to predicate the putative targets of *miR-34a-5p*. As shown in Figure 4A, we found that Smad4 was a potential target of *miR-34a-5p*, with the target site located in the 3′-UTR. It has previously been reported that Smad4 plays a significant role during invasion and migration in various cancers [17,18]. Moreover, *miR-34a-5p* reportedly target Smad4 in non-small-cell lung cancer (NSCLC) cells [14]. Thus, we considered demonstrating the possibility that *miR-34a-5p* also regulates the invasion and migration of trophoblast cells by targetting Smad4. To verify the potential targetting of Smad4 by *miR-34a-5p* in trophoblast cells, we constructed wild-type and mutant firefly luciferase reporters containing the 3′-UTR of Smad4. The reporters were co-transfected with either *miR-34a-5p* mimics/inhibitor or with NC mimics/inhibitor into HTR8/SVneo cells, and luciferase activity was then measured. We observed that overexpression of *miR-34a-5p* decreased relative luciferase activity of HTR8/SVneo cells in the presence of the wild-type 3′-UTR, whereas knockdown of *miR-34a-5p* increased the relative luciferase activity (Figure 4B). However, the luciferase activity did not changed significantly when the targetted sequence of Smad4 was mutated in the *miR-34a-5p*-binding site. To further validate the association between Smad4 and *miR-34a-5p*, we quantified the level of Smad4 mRNA and protein expression in HTR8/SVneo cells was analyzed by qRT-PCR and Western blot analysis. We found that the mRNA and protein levels of smad4 were significantly down-regulated after transfection of HTR8/SVneo cells with *miR-34a-5p* mimics, while transfection with *miR-34a-5p* inhibitor enhanced the mRNA and protein levels of Smad4 (Figure 4C, D). These data indicate that Smad4 is a target of *miR-34a-5p* in human trophoblast cells.

### Inverse correlation of *miR-34a-5p* and Smad4 expression levels in PE clinical samples

**Figure 5:**
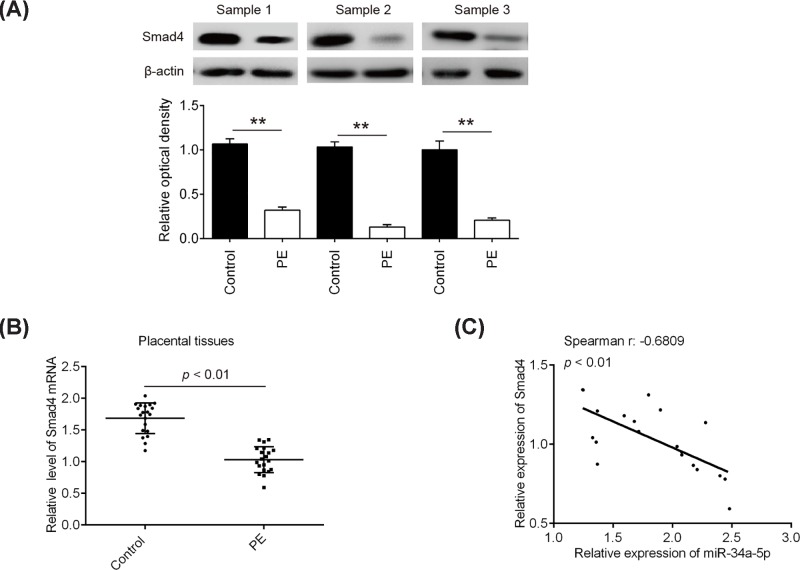
Inverse correlation of *miR-34a-5p* and Smad4 expression levels in PE clinical samples (**A**) The expressions of Smad4 protein were determined by Western Blot. The representative images of three pair tissues were presented. Bands were quantitatively compared between groups.^**^P<0.01 vs. Control group. (**B**) The expression levels of Smad4 were measured by Real-time PCR. *P*<0.01 vs. control group. (**C**) The relationship between *miR-34a-5p* and Smad4 was analyzed by Pearson’s or Spearman’s analysis. (r = −0.6809, *P*<0.001)

In order to further elucidate the correlation between Smad4 and *miR-34a-5p* expression, we measured the expression of Smad4 protein by Western Blot in 20 normal placentas and 20 severe PE placentas samples used here. The representative images showed that the expression levels of Smad4 protein were found to be significantly down-regulated in severe PE placentas, compared with normal placentas (Figure 5A). Similarly, its mRNA expression level was also reduced in severe PE placentas examined by qRT-PCR (Figure 5B). As expected, Spearman’s rank correlation analysis revealed that Smad4 expression was inversely correlated with *miR-34a-5p* expression in placentas tissues (r = −0.6809, *P*<0.001) (Figure 5C). Taken together, these findings indicate that *miR-34a-5p* may also inhibit the expression of Smad4 in placentas tissues, suggesting that *miR-34a-5p*/Smad4 axis may be involved in the mediation of the invasion and migration of trophoblast cells.

### Down-regulation of *miR-34a-5p* promoted the invasion and migration of trophoblast cells by targetting Smad4

**Figure 6:**
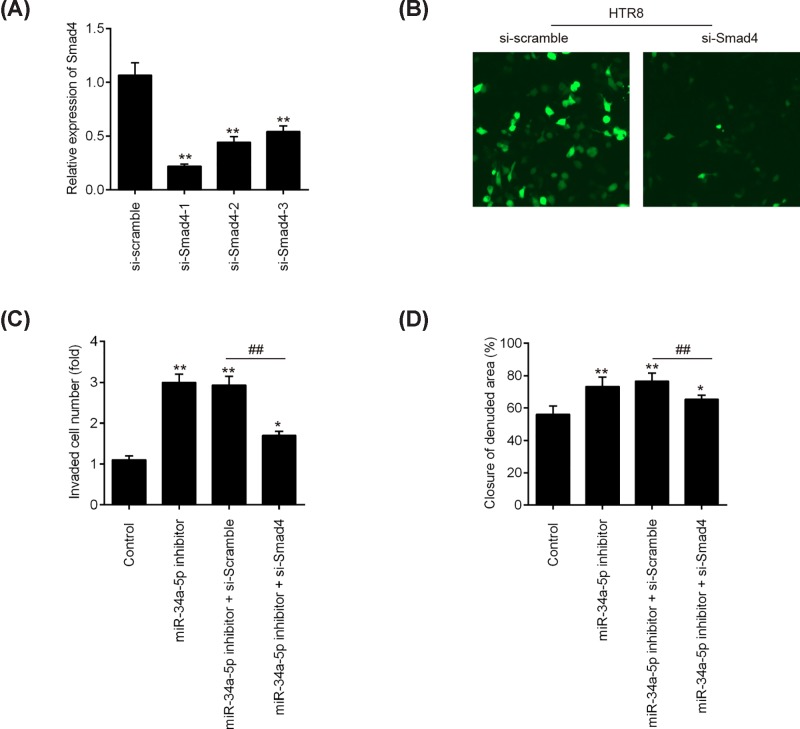
Down-regulation of *miR-34a-5p* promoted the invasion and migration of trophoblast cells by targetting Smad4 (**A**) The expression of Smad4 was measured by qRT-PCR after si-Smad4-1, si-Smad4-2, and si-Smad4-3 transfection in HTR-8/SVneo cells. Data represent the mean ± S.D. of three independent experiments. ^**^P<0.01 vs si-Scramble. (**B**) The expression of Smad4 protein was determined by immunofluorescence after si-Smad4 transfection in HTR-8/SVneo cells. (**C** and **D**) Cell migration and migration were measured using the transwell assay and wound healing assay, respectively. HTR-8/SVneo cells were co-transfected with *miR-34a-5p* inhibitor and si-Smad4 for 24 h.

To verify whether the function of *miR-34a-5p* is exerted via regulation of Smad4, we silenced the endogenous Smad4 expression using three specific siRNAs in HTR8/SVneo cells. As shown in Figure 6A, si-Smad4-1, si-Smad4-2, and si-Smad4-3 could effectively reduce the expression of Smad4 mRNA, especially si-Smad4-1. The expression of Smad4 was also evaluated at the protein level by Immunofluorescence assay, and we found the protein level of smad4 was also significantly down-regulated after transfection of si-Smad4 (Figure 6B). Subsequently, we co-transfected HTR8/SVneo cells with si-Smad4-1 and *miR-34a-5p* inhibitor to perform a rescue experiment. As shown in Figure 6C and D, knockdown of Smad4 expression attenuated the promoting effects of the *miR-34a-5p* inhibitor on migration and invasion in HTR8/SVneo cells. Above data suggest that down-regulation of *miR-34a-5p* promotes trophoblast cells invasion and migration, at least in part, via up-regulating Smad4.

## Discussion

In the present study, we found that *miR-34a-5p* was up-regulated in placental tissues and serum of patients with severe PE. We also proved that *miR-34a-5p* inhibition could enhance the invasion and migration of trophoblast cells via targetting Smad4 using *in vitro* functional and rescue experiments. Our results highlight down-regulation of *miR-34a-5p* may as a novel therapeutic strategy for PE.

Accumulating evidence has suggested that miRNAs play crucial regulatory roles in pregnancy-related diseases [19–21], including PE [22–24]. For example, up-regulation of *miR-203* contributed to PE through its inhibitory effects on invasion and migration of trophoblast cells [25]. Similar results have been demonstrated in studies investigating *miR-136* [26] and *miR-125b* [27], which also contribute to the development of PE by influencing the invasion and migration of trophoblast cells. In the present study, microarray screening revealed that *miR-34a-5p* to be one of the major miRNAs that were up-regulated in the placentas of patients with PE, which was consistent with previous reports [28,29]. Although *miR-34a-5p* has been extensively studied in the aspects of tumor cells invasion and metastasis in different cancers [30,31], the role of *miR-34a-5p* in the pathophysiology of PE has been rarely reported. Trophoblast cell invasive ability seems to contribute to the pathogenesis of PE [5]. Cumulative evidence indicates that miRNAs are implicated in regulating these events, providing new sight into the understanding trophoblast cell invasion and migration [32]. In the present study, we found that overexpression of *miR-34a-5p* decreased the cell invasive ability, while knockdown of *miR-34a-5p* led to the promotion of trophoblastic cells migration and invasion, suggesting that *miR-34a-5p* inhibition may improve inadequate trophoblast invasion in PE patients.

Smad4, as the common-partner Smad, is a pivotal mediator of transforming growth factor-β (TGF-β) signaling pathway and could play a significant role in tumor invasion and metastasis [33]. For example, Ding et al. found that loss of Smad4 inhibits pancreatic tumor cell metastases and plays a key regulator role in pancreatic tumor progression in mice and humans [34]. Lee et al. showed that inhibition of Smad4 protein expression by the aryl hydrocarbon receptor (AhR) suppressed tumor metastasis via epithelial to mesenchymal transition (EMT) reduction in lung cancer cells [35]. Notably, several studies have demonstrated that Smad4 exerts inhibitory effects on the invasion of trophoblast cells. For example, Li et al. found that depletion of Smad4 inhibited activin A, B, and AB-induced human trophoblast cell invasion through down-regulation of N-cadherin in HTR8/SVneo cells [36]. Lin et al. demonstrated that Smad4 up-regulated the expression of matrix metalloproteinases-2 (MMP-2) and elevated MMP2 subsequently contributed to the promotion of human trophoblast cell invasion [37]. Given the importance of Smad4 and *miR-34a-5p* in cells invasion and metastasis; herein, we sought to determine whether *miR-34a-5p* inhibition promotes the invasion and migration of trophoblastic cells through targetting Smad4. Based on the database and double-luciferase reporter assay, the results showed that *miR-34a-5p* can directly target Smad4. We further found that Smad4 was down-regulated in placental tissues from patients with severe PE, which is consistent with a previous study [38]. Moreover, the mRNA expression level of Smad4 was inversely correlated with *miR-34a-5p* levels in placental tissues. Importantly, knockdown of Smad4 reversed the promoting effects of *miR-34a-5p* inhibition on the invasion and migration of trophoblastic cells. All data suggest that *miR-34a-5p* inhibition improves inadequate trophoblast invasion through targetting Smad4.

In conclusion, we reported that *miR-34a-5p* expression was increased in placenta tissues from PE patients and knockdown of *miR-34a-5p* enhanced the invasion and migration of HTR-8/SVneo trophoblast cells through targetting Smad4. Our findings underlined the importance of *miR-34a-5p* in improving the invasion of trophoblast cells and presented a potential new therapeutic target against PE.

## Abbreviations

AhR: aryl hydrocarbon receptor
EMT: epithelial to mesenchymal transition
miRNA: microRNA
MMP2: matrix metalloproteinase 2
NC: negative control
NSCLC: non-small cell lung cancer
PE: preeclampsia
qRT-PCR: quantitative real-time PCR
SBP: systolic blood pressure
Smad: Small mothers against decapentaplegic
Smad4: Smad family member 4
TGF-β: transforming growth factor-β
UTR: untranslated region
